# Patterns of TIGIT Expression in Lymphatic Tissue, Inflammation, and Cancer

**DOI:** 10.1155/2019/5160565

**Published:** 2019-01-10

**Authors:** Niclas C. Blessin, Ronald Simon, Martina Kluth, Kristine Fischer, Claudia Hube-Magg, Wenchao Li, Georgia Makrypidi-Fraune, Björn Wellge, Tim Mandelkow, Nicolaus F. Debatin, Doris Höflmayer, Maximilian Lennartz, Guido Sauter, Jakob R. Izbicki, Sarah Minner, Franziska Büscheck, Ria Uhlig, David Dum, Till Krech, Andreas M. Luebke, Corinna Wittmer, Frank Jacobsen, Eike-Christian Burandt, Stefan Steurer, Waldemar Wilczak, Andrea Hinsch

**Affiliations:** ^1^Institute of Pathology, University Medical Centre Hamburg-Eppendorf, 20246 Hamburg, Germany; ^2^Dianova GmbH, Warburgstrasse 45, 20354 Hamburg, Germany; ^3^Department of General, Visceral and Thoracic Surgery, University Medical Centre Hamburg-Eppendorf, 20246 Hamburg, Germany

## Abstract

TIGIT is an inhibitory immune checkpoint receptor and a putative target for novel immune therapies. Here, we analysed two different types of tissue microarrays of healthy lymphatic and various inflamed tissues, colorectal and lung cancers, as well as >1700 tumour samples from 86 different tumour entities for TIGIT and/or PD-1 by bright field and/or multiplex fluorescence immunohistochemistry. TIGIT was detected in CD8^+^ cytotoxic T cells, CD4^+^ T helper cells, FOXP3^+^ regulatory T cells, and NK cells, but not in CD11c^+^ dendritic cells, CD68^+^ macrophages, and CD20^+^ B lymphocytes. TIGIT expression paralleled that of PD-1. More than 70% of TIGIT^+^ cells were PD-1^+^, and more than 90% of the PD-1^+^ cells were TIGIT^+^. Expression varied between different tissue compartments. TIGIT expression in tonsil gradually increased from the interfollicular area over the marginal/mantle zone to the germinal centre in all T cell subtypes. In inflammatory diseases, the strongest expression of TIGIT/PD-1 was found in Hashimoto thyroiditis. TIGIT^+^ lymphocytes were seen in all 86 different tumour entities with considerable high variability of TIGIT positivity within and between different cancer entities. Particularly, high densities of TIGIT^+^ lymphocytes were, for example, seen in squamous cell cancers of various origins. In summary, the variable expression levels of TIGIT and PD-1 in cell types and tissue compartments illustrate the high complexity of immune microenvironments. The high frequency of TIGIT (and PD-1) expressing lymphocytes in cancers highlights considerable opportunities for cotargeting with checkpoint inhibitors.

## 1. Introduction

Novel immune therapies using antibodies against immune checkpoint receptors, such as cytotoxic T lymphocyte antigen-4 (CTLA-4) and cell death protein-1 (PD-1), have demonstrated remarkable clinical efficiency in different tumour types, including metastatic melanoma, lung cancer, renal, and bladder carcinoma [1–3]. It is anticipated that blockade of other inhibitory immune checkpoint receptors will provide further therapeutic options.

T cell immunoglobulin and ITIM domain (TIGIT), a coinhibitory transmembrane glycoprotein of the poliovirus receptor (PVR) family, is another interesting checkpoint receptor. Only recently, it was suggested that anti-TIGIT drugs might be associated with less autoimmune-like toxicity, making TIGIT an appealing target for new cancer immunotherapies [4, 5]. TIGIT was first described as a T cell and natural killer (NK) cell-specific surface protein in 2009 [6–8]. TIGIT expression is restricted to T lymphocytes and highly expressed in effector and regulatory CD4^+^ T cells, follicular helper CD4^+^ T cells, effector CD8^+^ T cells, and NK cells [6, 7, 9–12]. Tumour infiltrating lymphocytes (TILs) expressing TIGIT have been demonstrated in several tumour types such as nonsmall cell lung cancer, colorectal carcinoma, melanoma, and acute myeloid leukaemia [9, 13, 14]. Although the downstream signalling cascade of TIGIT has not been clarified, there is evidence that TIGIT negatively regulates T cell activity through downregulation of T cell receptor expression [15–17]. In mouse models and on-going clinical studies, blockade or ablation of TIGIT, alone or in combination with blockade of PD-1, can restore tumour suppressive effects [4, 9, 13, 18, 19]. These findings indicate that TIGIT, similar as PD-1, has a crucial role in inhibiting the tumour-directed immune response and, thus, might be a suitable and relevant target for novel immune-modulating therapies. Several drugs targeting TIGIT are currently under development [20].

Previous studies on TIGIT were mostly limited to flow cytometer-sorted cells [6, 9, 13, 19, 21]. It is likely, however, that the expression of molecules regulating immune response does not only depend on the immune cell type but also on the tissue compartment where the immune cells are located. To study tissue compartment dependence of TIGIT expression in normal, inflammatory, and cancerous tissues, we made use of a new monoclonal antibody capable of detecting TIGIT in routine formalin-fixed tissue samples. The results of our study demonstrate high variability of TIGIT expression levels in inflammatory cells and frequent coexpression with PD-1 in normal, inflamed, and cancerous tissues.

## 2. Materials and Methods

### 2.1. Tissues

Two different types of tissue microarrays (TMAs) as well as large sections of HIV infected lymph nodes (*n* = 2), colorectal (*n* = 5), and lung cancers (*n* = 2) were analysed in this study. The first type of TMAs was “extra-large” microenvironment tissue microarrays (ME-TMAs). Formalin-fixed paraffin embedded tissue samples were selected from the archives of the Institute of Pathology of the University Medical Centre Hamburg-Eppendorf, Germany, for the construction of these arrays. The selection included normal lymph nodes (*n* = 3) and tonsils (*n* = 3), inflammatory diseases including Hashimoto thyroiditis (*n* = 10), sarcoidosis (*n* = 10), lichen sclerosus of the penis (*n* = 2), IgG4 pancreatitis (*n* = 2), and rheumatoid arthritis (*n* = 2). From all tissues, representative areas were selected for the construction of ME-TMAs specifically designed to study inflammatory responses. For optimal representation of the tissue microenvironment in the ME-TMA, a single tissue punch measuring 4 mm in diameter was taken from each donor. Selected areas included lymph follicles and surrounding tissue in healthy lymph node and tonsil, lymphocytic infiltrations, and adjacent epithelium in inflammatory conditions. Two different ME-TMAs were constructed, including a lymph node, tonsil, and inflammation ME-TMA (14 tissue spots (1 spot per donor) and 2 tissue spots from one tonsil sample as reference) and a ME-TMA from sarcoidosis and Hashimoto thyroiditis samples (10 tissue spots each (1 spot per donor) and 2 tissue spots from one tonsil sample as reference). Each ME-TMA contained two punches of the same tonsil as reference tissue for normalization of the measurement of fluorescence staining intensities. The second type of TMA was a multitumour array featuring 0.6 mm spots, which has been described before [22]. In brief, the multitumour TMA contained 3899 primary tumours from 99 different tumour types and subtypes distributed among 10 different TMA blocks each containing between 350 and 680 samples. A detailed list of the analysable cancers is given in the results section. The usage of archived diagnostic leftover tissues for manufacturing of tissue microarrays and their analysis for research purposes as well as patient data analysis has been approved by the local laws (HmbKHG, §12a) and by the local ethics committee (Ethics commission Hamburg, WF-049/09).

### 2.2. Bright Field and Fluorescence Immunohistochemistry

Freshly cut 4 *μ*m consecutive tissue sections were used for immunohistochemistry (IHC) analyses. Specificity of the anti-TIGIT antibody clone TG1 (Dianova, Hamburg, Germany) was validated by Western blot, ELISA, and preabsorption of the primary antibody with TIGIT protein.

For bright field IHC, tissue sections were dewaxed and incubated in antigen retrieval solutions (Table 1) prior to blocking of endogenous peroxidase and incubation of the primary and secondary antibodies. Bound antibody was detected with the DAB kit (DAKO, Santa Clara, United States), and slides were counterstained and sealed in Eukit. Dilution series were performed in individual experiments to validate perceived expression differences on ME-TMAs.

For fluorescence multiplex IHC, the OPAL dye kit (Cat. #OP7DS1001KT, Perkin Elmer, Waltham, Massachusetts, United States) was used. Details on the used antibodies, antibody retrieval procedures, and OPAL dyes are given in Table 1. The experimental procedure was performed according to the manufacturer's instructions [23]. Slides were initially boiled in a microwave (15 minutes at 100°C) for antigen retrieval. Three different primary antibodies were combined with DAPI staining in each experiment. One circle of antibody staining included peroxidase blocking, application of the primary antibody, detection with a secondary HRP-conjugated antibody, fluorescence dye detection, and removal of the bound antibodies by microwave treatment (15 minutes at 100°C). This cycle was repeated two times for the remaining antibodies. Slides were subsequently counterstained with diamidinoino-2-phenylindole (DAPI) and mounted in an antifade solution.

### 2.3. Analysis of Bright Field and Fluorescence Staining

Conventional bright field staining was used to quantify the density of TIGIT^+^ cells in individual TMA cores of the multitumour TMA. All multitumour TMA slides were visually inspected under a microscope. The number of TIGIT^+^ cells per 0.6 mm tissue spot was manually counted and converted into the density of TIGIT^+^ cells per square mm. All slides stained with fluorescent dyes were scanned using Leica's Aperio VERSA 8 automated epifluorescence microscope. A pathologist defined tissue compartments (e.g., tumour area, stromal area, lymphocytic infiltrations, germinal centres, and marginal zones of lymph follicles) with sufficient numbers of leucocytes (approx. 800 to 6000 cells) for digital image analysis using the ImageScope software package (Leica Microsystems, Wetzlar, Germany). The intensity of each fluorochrome in each individual cell (i.e., a continuous numerical value indicating the fluorescence signal strength) was recorded as raw intensity data. Only staining intensities exceeding a predefined threshold were considered “positive”. The threshold was individually selected for each marker (CD3, CD4, CD8, FOXP3, CD11c, CD56, CD68, PD-1, and TIGIT) according to the following procedure: the fluorescence intensity of each marker was measured in 50 to 200 cells with expected lack of expression, and the value of the cell with highest “false positive” measurement was used to define the cut-off value for positive expression. For comparison of expression levels of TIGIT and PD-1 in the defined tissue compartments, we normalized our measurements to reference cells. As reference cells, we used the cells located within the germinal centres of normal tonsil tissues, which showed the highest expression levels of TIGIT and PD-1 across all healthy human tissues tested. Accordingly, we placed tonsil tissue as a reference on each slide, and the mean raw intensity of the fluorochromes associated with TIGIT and PD-1 in the germinal centres was set to 100%. The relative expression (RE) was then calculated as the percentage of mean raw intensity of the test cells in relation to the 100% mean raw intensity in the reference cells.

### 2.4. Statistical Analysis

JMP Pro 12 software package (SAS Institute Inc., NC, USA) and R version 3.4.3 (the R foundation) [24] were used to plot the data, to perform analysis of variance (ANOVA), and to calculate compartment-specific expression differences of PD-1 and TIGIT.

## 3. Results

### 3.1. Patterns of TIGIT Expression in Lymph Nodes and Tonsils

**Figure 1:**
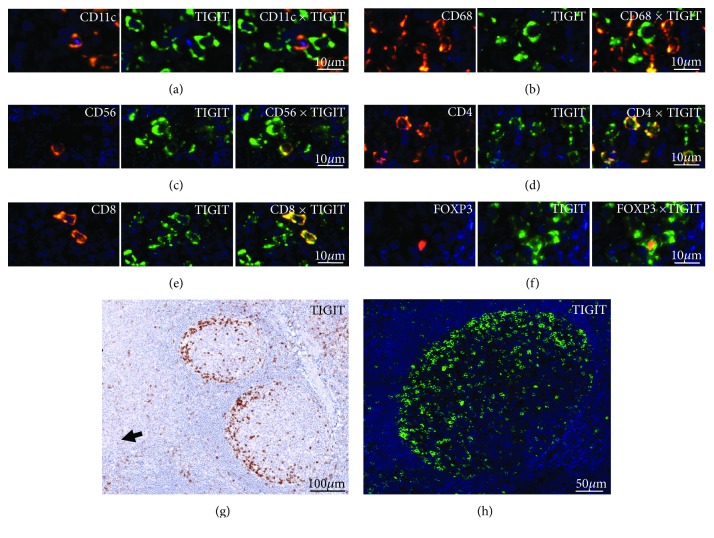
Representative pictures of TIGIT staining in human tonsils by multiplex immunohistochemistry in combination with (a) CD4, (b) CD8, (c) FOXP3, (d) CD56, (e) CD11c, and (f) CD68. (g) Bright field image and (h) fluorescence photograph showing TIGIT staining at the periphery of the germinal centre. Note the orientation of the stained cells towards the loosened epithelium of the tonsil (arrowhead).

**Figure 2:**
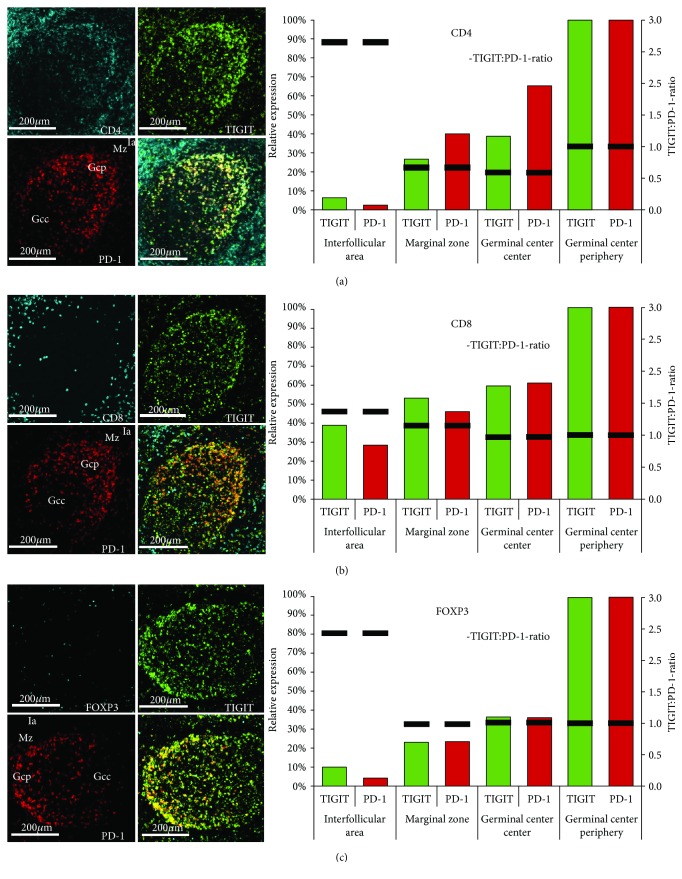
Intensity differences of TIGIT (green) and PD-1 (red) expression in (a) CD4^+^ helper T cells, (b) CD8+ cytotoxic T cells, and (c) FOXP3^+^ regulatory T cells between the interfollicular area (Ia), the marginal zone (Mz), the germinal centre periphery (Gcp), and the germinal centre (Gcc) of human tonsils. Relative expression refers to the fluorescence measurement in the tonsil germinal centre periphery set to 100%. Columns indicate the relative expression levels of TIGIT (green) and PD-1 (red). The black bar shows the TIGIT : PD-1 expression ratio.

In lymph nodes and tonsils, TIGIT^+^ cells were seen in the interfollicular area, the marginal/mantle zone surrounding lymph follicles, and the germinal centre. Multiplex immunofluorescence analysis of TIGT on CD20^+^ B lymphocytes, CD3^+^ T lymphocytes, various T cell subtypes (CD4^+^, CD8^+^, FOXP3^+^; Figures 1(a)–1(c)), CD56^+^ natural killer cells (Figure 1(d)), CD11c^+^ dendritic cells (Figure 1(e)), and CD68^+^ macrophages (Figure 1(f)) revealed that TIGIT expression is most frequently detected in T lymphocytes and also in a subset of NK-cells. At the selected threshold, 52% of CD3^+^ T cells were TIGIT positive, while no unequivocal staining was seen in CD20^+^ lymphocytes. TIGIT positivity was detected in 47% of CD4, 53% of CD8, and 72% of FOXP3^+^ T cells. The highest level of TIGIT expression was found in T cells located in the germinal centre periphery orientated towards the tonsil surface epithelium (Figures 1(g) and 1(h)). These were predominantly CD4^+^ follicular T helper cells but also T cells of other subtypes (i.e., CD8 and FOXP3). Second highest levels of TIGIT were found in CD8^+^ cytotoxic lymphocytes located in the interfollicular area. Overall, TIGIT expression gradually increased from the interfollicular area over the marginal/mantle zone to the germinal centre in all T cell subtypes, particularly in CD4^+^ and FOXP3^+^ T cells (Figure 2). Also, the fraction of TIGIT^+^ CD4^+^ cells varied markedly between these compartments; while the vast majority (>95%) of CD4^+^ T cells in the lymph follicles showed TIGIT expression, this was true for only about 47% of the CD4^+^ T cells in the interfollicular compartment.

### 3.2. Relationship between TIGIT and PD-1 in Lymph Node and Tonsils

Expression patterns of TIGIT and PD-1 were highly congruent; more than 70% of all TIGIT^+^ cells also expressed PD-1, and more than 90% of the PD-1 positive cells were also TIGIT positive. Similarly, as for TIGIT, there was a strong increase of PD-1 expression levels from outside to inside of the lymph follicles (Figure 2). Expression of both proteins was therefore jointly assessed (and compared) for subsequent analyses. For this purpose, MF-IHC analysis was performed for each cell type of interest (CD4^+^, CD8^+^, and FOXP3^+^), and the findings were recorded for every individual tissue compartment (interfollicular area, marginal/mantle zone, and germinal centre; Figure 2). Interestingly, these analyses revealed differences in the TIGIT : PD-1 expression ratio depending on the T cell type and tissue compartment. While the TIGIT : PD-1 ratio in CD8^+^ T cells remained constant inside and outside the germinal centres (Figure 2(b)), this ratio changed in CD4^+^ (Figure 2(a)) and FOXP3^+^ (Figure 2(c)) T cells towards higher relative expression of TIGIT outside the germinal centres.

### 3.3. TIGIT and PD-1 in Lymph Nodes of Patients with Human Immunodeficiency Virus (HIV) Infection

Patients in early phase HIV infection often have severe follicular hyperplasia in their lymph nodes [25]. These morphological changes were accompanied by a loss of the characteristic orientation of TIGIT^+^ and PD-1^+^ T helper cells towards the lymph node surface. Instead, TIGIT/PD-1 positive CD4^+^ T cells were evenly distributed across the germinal centre in two analysed lymph nodes from HIV-infected patients. However, fluorescence measurements and serial dilutions revealed that the architectural changes had no impact on the expression levels of TIGIT and PD-1, since these were comparable to the staining levels found in normal tonsil and lymph node (Supplementary Figure S1).

### 3.4. TIGIT and PD-1 in Hashimoto Thyroiditis and Sarcoidosis

**Figure 3:**
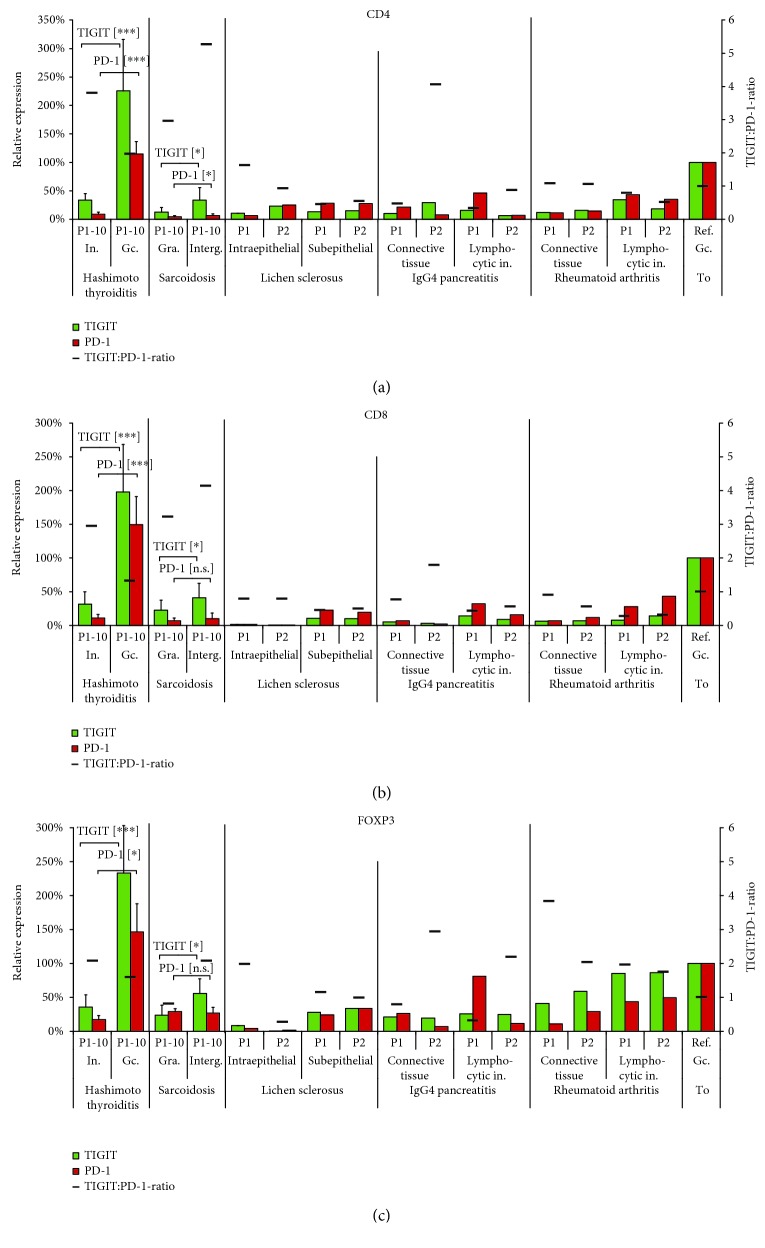
Interindividual variations of TIGIT (green) and PD-1 (red) in 10 patients (P1-10) each with Hashimoto thyroiditis and sarcoidosis; two patients (P1 and P2) each with lichen sclerosus, IgG4 pancreatitis, and rheumatoid arthritis of (a) CD4^+^ helper T cells, (b) CD8^+^ cytotoxic T cells, and (c) FOXP3^+^ regulatory T cells. Relative expression refers to the fluorescence measurement in the tonsil germinal centre periphery (Ref. Gc. To.) set to 100%. The black bar shows the TIGIT : PD-1 expression ratio. In.: interfollicular area; Gc.: germinal centre; Gra.: granuloma; Interg.: intergranuloma; Lymphocytic in.: lymphocytic infiltration; To: tonsil; ^∗^ *p* ≤ 0.05; ^∗∗^ *p* ≤ 0.001; ^∗∗∗^ *p* ≤ 0.0001; [n.s.]: not significant (*p* > 0.05).

We selected Hashimoto thyroiditis and sarcoidosis because they reflect inflammatory conditions associated with germinal centre formation (thyroiditis) or destruction (sarcoidosis). Strikingly, the highest levels of TIGIT and PD-1 expression among all tissues included in this study were found in lymph follicles of Hashimoto thyroiditis. Fluorescence measurements in 10 cases revealed that the expression level of TIGIT and PD-1 was about 2-3 times higher than in the comparable areas of lymph follicles in normal tonsil or lymph node. This applied for all three T cell subtypes analysed (Figure 3). The strikingly high TIGIT and PD-1 expression was confirmed by conventional bright field IHC analysis employing serial dilutions of the primary antibodies (Supplementary Figure S2 and S3). In contrast to the largely balanced TIGIT : PD-1 expression ratio in thyroiditis, sarcoidosis was characterized by relative overexpression of TIGIT. In sarcoidosis, fluorescence measurement further revealed compartment-specific differences of TIGIT, but not of PD-1 expression; TIGIT levels were significantly higher in T cells (including CD4^+^, CD8^+^, and FOXP3^+^) in the intergranulomatous area as compared to the granulomas (*p* < 0.05, Figure 3). It is of note that both Hashimoto thyroiditis and sarcoidosis showed only little variation of the TIGIT and PD-1 expression levels between the 10 analysed individuals each (Supplementary Figure S4).

### 3.5. TIGIT and PD-1 in Other Types of Inflammation

The analyses of two cases each of selected inflammatory diseases, including lichen sclerosus, IgG4-pancreatitis, and rheumatoid arthritis revealed detectable TIGIT and PD-1 expression in all analysed samples (Figure 3). Also in these conditions expression of TIGIT largely paralleled the pattern of PD-1 expression. A comparison of the normalized TIGIT and PD-1 expression levels in CD4^+^, CD8^+^, and FOXP3^+^ cells of different types of inflammation is given in Figure 3. In all these samples, it appeared that the expression of TIGIT and PD-1 depended on the T cell density. In lymphocyte-dense compartments, such as areas of lymphocytic infiltration in IgG4 pancreatitis or rheumatoid arthritis, levels of TIGIT and PD-1 were higher than in areas containing fewer and scattered lymphocytes. Interestingly, PD-1 appeared to be more highly upregulated than TIGIT in the lymphocyte-rich areas of most inflammations (except sarcoidosis), as indicated by the TIGIT : PD-1 ratio < 1.0.

### 3.6. TIGIT Expression in Human Cancers

**Figure 4:**
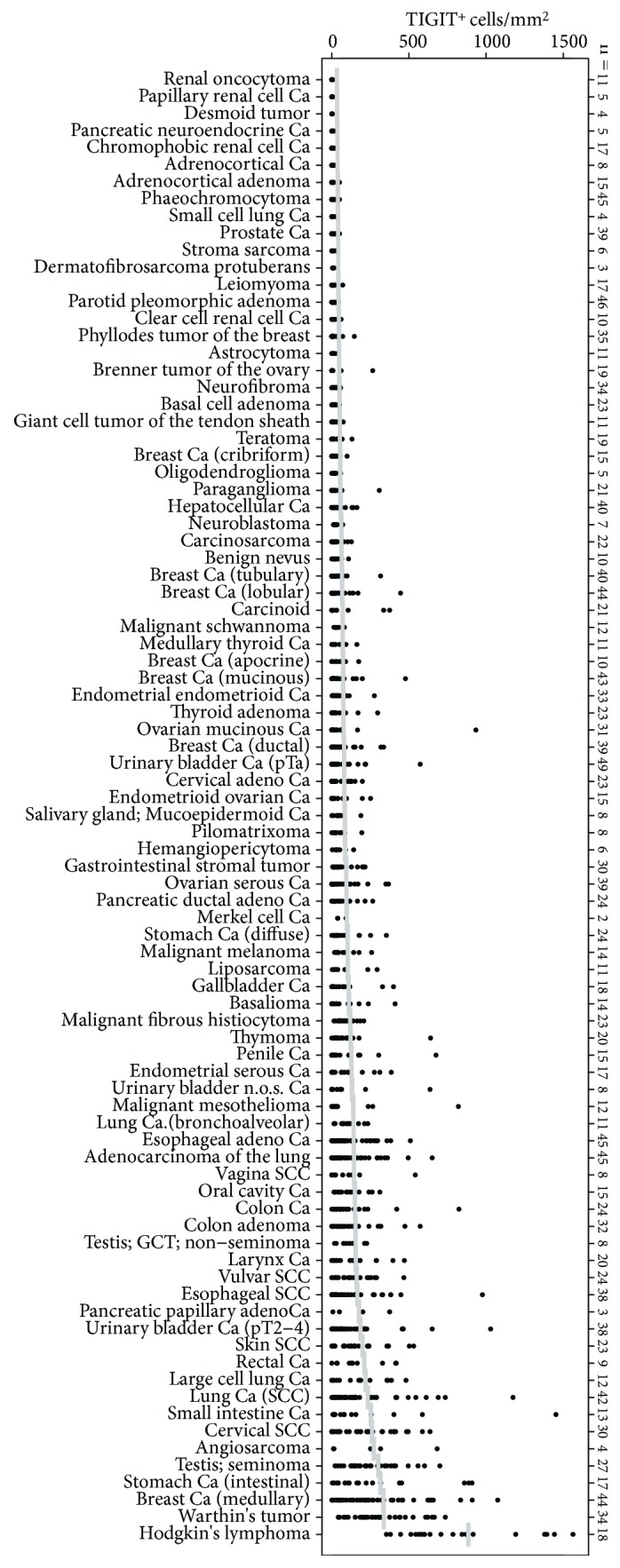
Distribution of the cell density of TIGIT^+^ cells (cell/mm^2^) across 86 human tumour entities. In total, 1778 tumour samples, represented by the dots, have been analysed. Horizontal bars indicate the average density per entity. Ca: carcinoma; SCC: squamous cell carcinoma; GCT: germ cell tumour.

Interpretable results could be obtained from 86 of the 99 cancer types represented in our multitumour TMA. All interpretable cancer types had at least one case with TIGIT^+^ lymphocytes. The density of TIGIT^+^ T cells was highly variable within all analysed cancer types. Considerable differences were found, however, in the average number of TIGIT^+^ cells per cancer entity. As expected, the highest densities of TIGIT^+^ lymphocytes were found in tumours characterized by high numbers of tumour-associated lymphocytes such as Hodgkin's lymphoma (average: 852 ± 380 cells/mm^2^), Warthin's tumours (average: 305 ± 195 cells/mm^2^), medullary breast cancer (average: 302 ± 363 cells/mm^2^), or seminoma (average: 268 ± 177 cells/mm^2^). Other cancers with particularly high fractions of TIGIT^+^ cells included intestinal stomach cancer (average: 283 ± 316 cells/mm^2^) and squamous cell cancers of various origins (average 228-112 cells/mm^2^). Tumour types with lowest TIGIT^+^ lymphocyte densities were renal oncocytoma, papillary and chromophobic renal cell cancer, desmoid tumour, neuroendocrine pancreatic cancer, and cancer of the adrenal cortex (average all <6 cells/mm^2^). All data are summarized in Figure 4.

### 3.7. T Cell Density and TIGIT/PD-1 Expression in Colorectal and Lung Cancers

**Figure 5:**
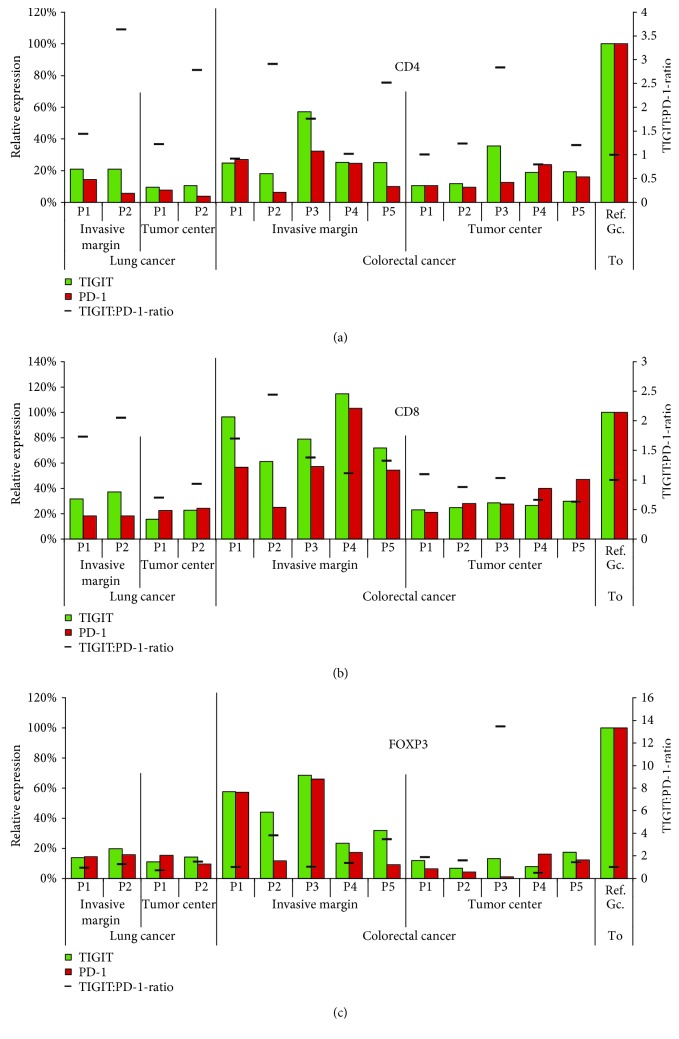
Interindividual variations of TIGIT (green) and PD-1 (red) of (a) CD4^+^ helper T cells, (b) CD8^+^ cytotoxic T cells, and (c) FOXP3^+^ regulatory T cells among two patients (P1 and P2) with lung cancer and five patients (P1-5) with colorectal cancer. Relative expression refers to the fluorescence measurement in the tonsil germinal centre periphery (Ref. Gc. To.) set to 100%. The black bar shows the TIGIT : PD-1 expression ratio.

**Figure 6:**
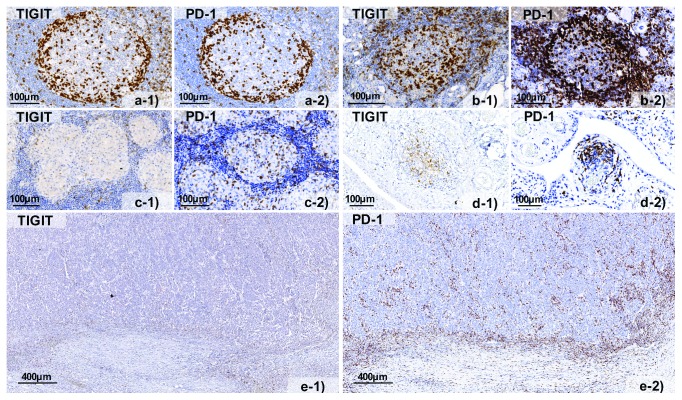
Representative pictures of TIGIT (−1) and PD-1 (−2) staining in (a) normal human tonsil, (b) Hashimoto thyroiditis, (c) sarcoidosis, (d) rheumatoid arthritis, and (e) colorectal cancer.

To study the differences of TIGIT expression between the invasive margin and the tumour centre, we selected large sections of lung (2 cases) and colorectal cancer (5 cases) for multiplex fluorescence immunohistochemistry. There was a markedly higher density of TIGIT^+^ T cells (e.g., 59 ± 49 CD8^+^TIGIT^+^ cells per 0.1 mm^2^) at the invasive margin as compared to the tumour centre (10 ± 13 cells per 0.1 mm^2^) for almost all analysed cancers. PD-1 was included to search for coexpression patterns. Comparison of TIGIT expression levels with that of PD-1 revealed a relative predominance of TIGIT or PD-1, which varied between individual cancers. The TIGIT : PD-1 ratio ranged between 0.75 and 4.0 in individual cases. Moreover, compartment-specific differences were also found for TIGIT and PD-1 expression levels in these tumours. In colorectal cancers, expression of TIGIT and PD-1 was considerably higher in T cells located at the invasive margin as compared to T cells in the tumour centre. This was particularly true for CD8^+^ lymphocytes, which showed the highest TIGIT and PD-1 expression levels on average (Figure 5). Representative images showing TIGIT and PD-1 expression by bright field immunohistochemistry in the examined tissues are given in Figure 6.

## 4. Discussion

Many previous studies on TIGIT were limited by the lack of antibodies suitable for detecting TIGIT in routinely formalin-fixed paraffin embedded tissues. Consequently, current knowledge on TIGIT expression in human immunological tissues comes almost exclusively from the analysis of disintegrated tissues by means of flow cytometry or mRNA analysis [6, 9, 13, 19, 21]. The data from this study demonstrate that TIGIT expression varies between tissue compartments and cell types in normal lymphatic tissues and various inflammatory and cancerous conditions.

Our analysis of a variety of normal and inflamed human tissues identified variable fractions of TIGIT expressing CD8^+^ cytotoxic T cells, CD4^+^ T helper cells, FOXP3^+^ regulatory T cells, and NK cells, while unequivocal TIGIT staining was not seen in CD11c^+^ dendritic cells, CD68^+^ macrophages, and CD20^+^ B lymphocytes. The limitation of TIGIT expression to specific leucocyte subtypes is in line with earlier data [15, 26] and confirms the validity of our multiplex immunohistochemistry approach.

The strong overlap of TIGIT and PD-1 expands the results of earlier studies demonstrating the coexpression of both proteins [9, 13], but also other checkpoint receptors such as Lag3 [27] and Tim3 [28, 29], in tumour infiltrating lymphocytes and is consistent with recent reports describing comparable properties for the TIGIT/CD155/CD226 regulatory pathway and the PD-1/PD-L1-immune checkpoint [16, 17, 30, 31]. Both PD-1 and TIGIT are increasingly upregulated in activated T lymphocytes, most likely to prevent overly excessive immune responses [15, 32].

The most significant finding of our study was that expression levels of both TIGIT and PD-1 varied not only according to cell types but also according to the cellular localization and context. In some instances, tissue compartment-specific TIGIT and/or PD-1 expression patterns may be linked to specific cell functions. The striking polar expression of TIGIT and PD-1 in normal lymph node and tonsil colocalizes with the “light zone” of the lymph follicle, where T helper cell mediated B-cell maturation and immunoglobulin class switching occurs [33]. It is intuitive that the expression of TIGIT (and PD-1) in these follicular T helper cells is part of the regulation network fine-tuning T cell activity. Distortion of the polar orientation of TIGIT^+^ and PD-1^+^ follicular T helper cells in lymph follicles from HIV-infected patients may indicate lymphocyte dysfunction. This finding fits well to the concept that T helper cells inside the germinal centres represent a reservoir for HIV infection where they are shielded from engagement by cytotoxic T cells [34]. It has been shown that these HIV-infected T cells express high levels of PD-1 [35].

Inflammatory tissues do not only provide a valuable microenvironment for T cell research but also represent the site of immunotherapy-related adverse events. The number of inflammatory conditions analysed in this study was limited, but it was conspicuous that the ratio between TIGIT and PD-1 expression was comparable in most types of inflammations. This might suggest that the interplay between these pathways is rather similar across different types and causes of inflammation. The generally higher expression of TIGIT and PD-1 in lymphocyte-dense areas of inflammatory diseases than in less densely populated areas (i.e., connective tissues, intraepithelial areas) fits well to the concept of compensatory downregulation of excessive inflammatory reactions through immune checkpoint upregulation [36, 37]. It is of note that among all analysed tissues, the highest levels of TIGIT (and PD-1) expression were constantly seen on T helper cells in lymph follicles of Hashimoto thyroiditis. Thyroiditis is the most frequent endocrine immune-related adverse event of anti-PD-1 therapy [38]. A recently published study analysing anti-PD-1 therapy-induced Hashimoto thyroiditis describes an increased fraction of follicular T helper cells in peripheral blood [39]. It is possible that these T helper cells origin from the germinal centres of Hashimoto thyroiditis. Sarcoidosis differed from all other inflammatory conditions analysed in this study in its higher relative levels of TIGIT as compared to PD-1. This observation suggests that the relative role of individual checkpoint molecules may considerably vary between different inflammatory diseases. It will be interesting to see whether expression patterns of immunoregulatory proteins may have diagnostic utility and perhaps assist treatment decisions in some inflammatory diseases in the future.

Finding TIGIT^+^ lymphocytes in all analysed 86 different tumour entities in an analysis of 1700 individual cancer tissues identified TIGIT expression as a general feature of tumour-associated lymphocytes. As expected, there was a high variability of TIGIT positivity both within different samples of individual tumour types and also between different cancer entities. This variability parallels the variability seen in the number of tumour-infiltrating lymphocytes and may be due to differences in quantity and quality of immunogenic neoantigens [40, 41], variable mechanisms for immune evasion [42, 43], and probably other factors. A high variability of immune checkpoint proteins has been reported from a multitude of studies on different cancer types [44–46] and has eventually led to the definition of immune “-cold” and “-hot” cancer [47, 48]. That highest TIGIT^+^ lymphocyte density was found in tumours which are characterized by high number of lymphocytes such as Hodgkin's lymphoma, Warthin's tumour, seminoma, and medullary breast cancer that represents an indirect validation of our experimental approach. Other tumour types with conspicuously high densities of TIGIT^+^ lymphocytes included, for example, intestinal type stomach cancer and squamous cell cancers of various origins. It is tempting to speculate that these tumour types may benefit better from future anti-TIGIT therapies than those with lower amounts of TIGIT^+^ lymphocytes. However, our findings must be considered cautiously, because the 0.6 mm tissue cores of the used multitumour TMA may be too small for a reliable assessment of the lymphocytic infiltration in a tumour and its microenvironment.

To further explore the role of TIGIT in the tumour centre and at the invasive margin, we selected 7 cases of lung and colorectal cancers with representative tissue compartments. That the sparse and scattered CD8^+^ lymphocytes located in the tumour centre had lower levels of TIGIT (and PD-1) as compared to the densely clustered CD8^+^ lymphocytes at the invasive margin would again be consistent with the concept of compensatory upregulation of TIGIT and PD-1 in excessive inflammatory reactions. It cannot be excluded that these differences in the expression level indicate functional differences such as higher degrees of anticancer activity of tumour-infiltrating CD8^+^ cells as compared to CD8+ cells at the invasive tumour margin. Our findings support the idea that lymphocytes located at the invasive margin are the major targets of immune checkpoint therapies directed against the PD-1/PD-L1 axis [49–52], and that this may also hold true for potential future drugs targeting TIGIT. Several antibodies directed against TIGIT are currently being tested in phase I trials (OMP-31M32, MK-7684, AB154) and II (MTIG7192A, BMS-986207), but data on the clinical benefit are not available as to yet [4, 5, 20].

This study is an example for the importance of antibodies suitable for the analysis of formalin-fixed tissues. Our findings strongly support the view that it may be not only necessary to characterize the expression of multiple parameters per cell but also relevant to incorporate topographical aspects of inflammatory cells and their “target” cancer cells. For example, in a recent study on malignant melanomas, the proximity of the CD8^+^ PD-1^+^ cells and the PD-L1^+^ tumour cells at the invasive margin was associated with response to immune checkpoint inhibitors [49]. If novel therapies aiming at the immune environment of cancer hold their promises, the evaluation of the immune response to individual cancers may become a highly demanded routine application in diagnostic pathology. Multicolour imaging and image analysis systems will be indispensable for such analyses, although even the use of sophisticated image analysis systems does not prevent from analysis errors due to imperfect immunostaining. We thus used a tissue microarray approach to achieve the best possible standardization of the experimental parameters (including the reference tissue on the same slide), validated all relevant observations in this study by conventional bright field immunostainings, and used dilution series for confirmation of expression differences.

## 5. Conclusions

In summary, the data demonstrate that TIGIT expression is highly prevalent in T lymphocytes and that TIGIT expression largely parallels the expression pattern of PD-1. A simultaneous interruption of PD-1 and TIGIT signalling might thus have an additive effect on antitumour immunity. The variability of PD-1 and TIGIT expression between different cellular compartments underscores the importance of in situ analysis of patient tissues.

## Figures and Tables

**Table 1:** List of the used antibodies, antigen retrieval (AR), dilutions, and Opal dyes.

Antibody	Target	Bright field		Fluorescence			
		AR	Dilution	AR	Dilution	Order^1^	Dye
DAKO #IR503	CD3	pH 9	1 : 1	pH 9	1 : 1	1st	Opal 520
DAKO #IR649	CD4	pH 9	1 : 1	pH 9	1 : 1	1st	Opal 520
DAKO #IR623	CD8	pH 9	1 : 1	pH 9	1 : 1	1st^∗^	Opal 520^∗∗^
BioLegend #320102	FOXP3	pH 9	1 : 50	pH 9	1 : 50	1st	Opal 520
DAKO #IR604	CD20	pH 9	1 : 1	pH 9	1 : 10	1st^∗^	Opal 520^∗∗^
DAKO #IR613	CD68	pH 6	1 : 1	pH 6	1 : 1	1st	Opal 520
DAKO #IR628	CD56	pH 9	1 : 1	pH 9	1 : 1	1st	Opal 520
Abcam#ab52632	CD11c	pH 9	1 : 450	pH 9	1 : 450	1st	Opal 520
Dianova #DIA-TG1	TIGIT	pH 7.8	1 : 70	pH 9	1 : 150	2nd	Opal 570
Abcam #ab52587	PD-1	pH 6	1 : 50	pH 6	1 : 50	3rd	Opal 690

AR: antigen retrieval; ^1^order refers to the sequence of antibodies in multiplex fluorescence immunohistochemistry experiments; ^∗^antibody was used at third position when stained in combination with CD3, CD4; ^∗∗^with Opal 690 dye.

## Data Availability

The data used to support the findings of this study are available from the corresponding author upon request.

## Supplementary Materials

Supplementary 1 Figure S1: representative images at 100x magnification of lymph node staining in a healthy person and a HIV patient with serially diluted TIGIT antibody.

Supplementary 2 Figure S2: representative images at 100x magnification of a human tonsil and a thyroid gland with Hashimoto thyroiditis stained with serially diluted TIGIT antibody.

Supplementary 3 Figure S3: representative images at 100x magnification of a human tonsil and a thyroid gland with Hashimoto thyroiditis stained with serially diluted PD-1 antibody.

Supplementary 4 Figure S4: TIGIT (black) and PD1 (grey) expression levels in (a-c) sarcoidosis and (d-f) Hashimoto thyroiditis. Relative expression refers to the fluorescence measurement in the tonsil germinal centre periphery (Ref. Gc. To.) set to 100%. The black bar shows the TIGIT : PD-1 expression ratio. Gc.: germinal centre; To: tonsil; P: patient. Figure S4 TIGIT (black) and PD1 (grey) expression levels in (a-c) sarcoidosis and (d-f) Hashimoto thyroiditis. Relative expression refers to the fluorescence measurement in the tonsil germinal centre periphery (Ref. Gc. To.) set to 100%. The black bar shows the TIGIT : PD-1 expression ratio. Gc.: germinal centre; To: tonsil; P: patient.
