# Supplementary material for: Patterns of TIGIT Expression in Lymphatic Tissue, Inflammation, and Cancer
Source: Dis Markers. 2019 Jan 10;2019:5160565. doi: 10.1155/2019/5160565 (PMC6348838; doi:10.1155/2019/5160565)
Supplement: Supplementary 1 — Figure S1: representative images at 100x magnification of lymph node staining in a healthy person and a HIV patient with serially diluted TIGIT antibody. [file 5160565.f1.pptx]

## Slide 1
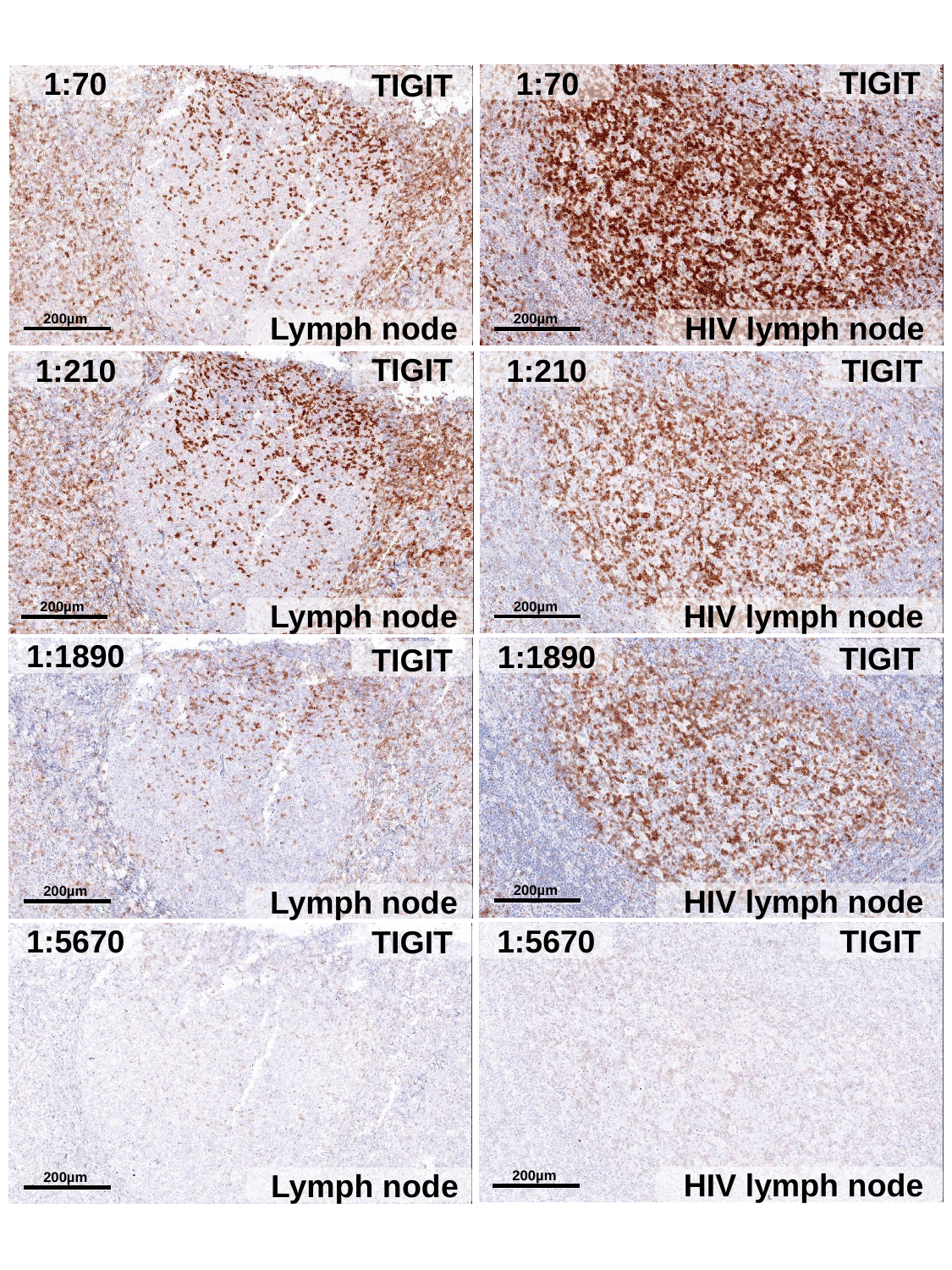

TIGIT
1:70
1:70
TIGIT
200µm
 Lymph node
 HIV lymph node
200µm
TIGIT
1:210
1:210
TIGIT
200µm
 HIV lymph node
 Lymph node
200µm
1:1890
1:1890
TIGIT
TIGIT
200µm
200µm
 HIV lymph node
 Lymph node
1:5670
1:5670
TIGIT
TIGIT
200µm
 HIV lymph node
 Lymph node
200µm
Supplementary Figure 2: Serial dilution of the TIGIT antibody in uninfected human lymph node and a lymph node of a HIV-patient to reevaluate the fluorescent results.
