# Supplementary material for: Patterns of TIGIT Expression in Lymphatic Tissue, Inflammation, and Cancer
Source: Dis Markers. 2019 Jan 10;2019:5160565. doi: 10.1155/2019/5160565 (PMC6348838; doi:10.1155/2019/5160565)
Supplement: Supplementary 2 — Figure S2: representative images at 100x magnification of a human tonsil and a thyroid gland with Hashimoto thyroiditis stained with serially diluted TIGIT antibody. [file 5160565.f2.pptx]

## Slide 1
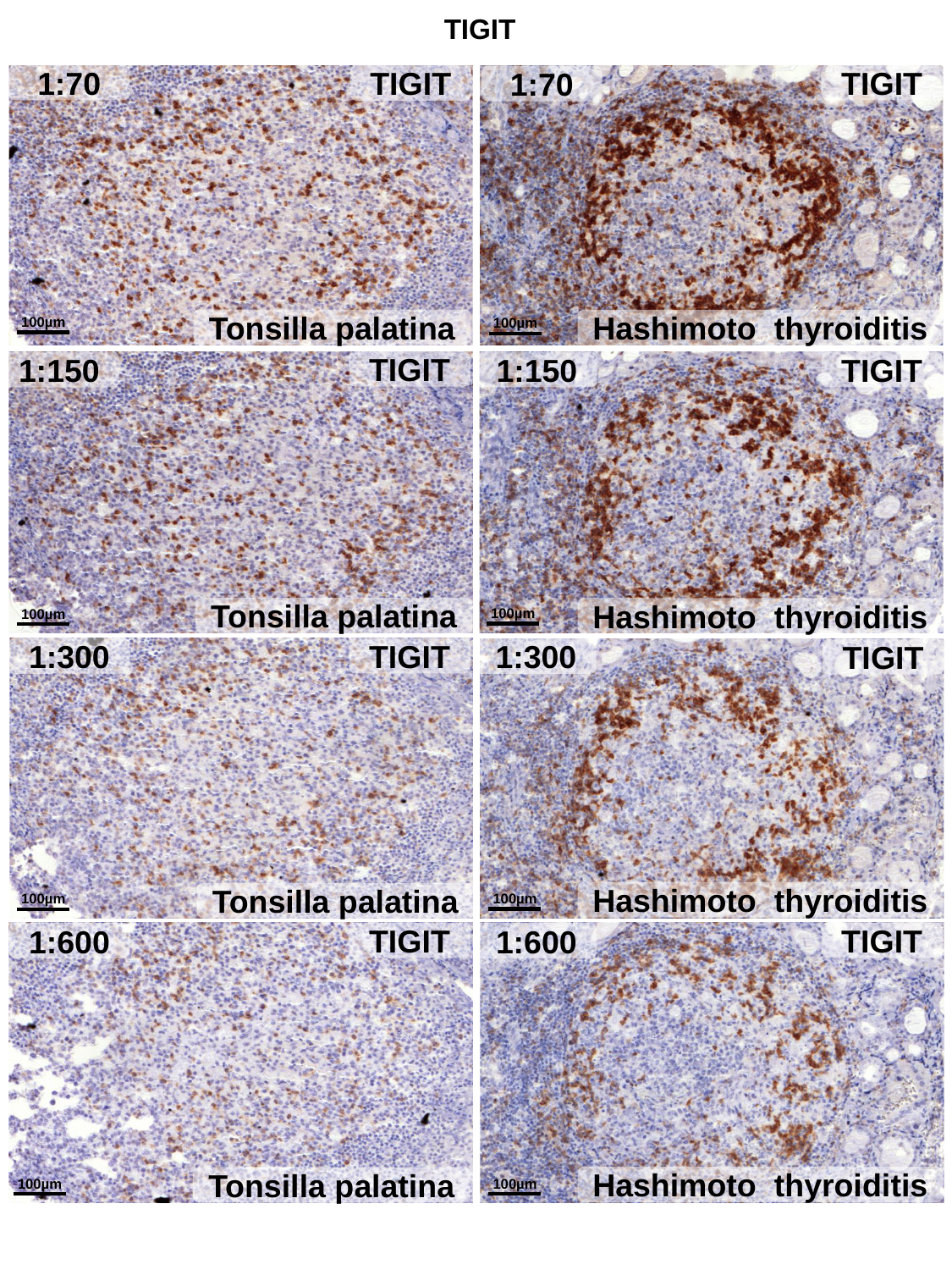

TIGIT
1:70
TIGIT
TIGIT
1:70
Tonsilla palatina
Hashimoto  thyroiditis
100µm
100µm
TIGIT
1:150
1:150
TIGIT
Tonsilla palatina
Hashimoto  thyroiditis
100µm
100µm
1:300
TIGIT
1:300
TIGIT
Hashimoto  thyroiditis
Tonsilla palatina
100µm
100µm
TIGIT
TIGIT
1:600
1:600
Hashimoto  thyroiditis
Tonsilla palatina
100µm
100µm
