# Supplementary material for: Patterns of TIGIT Expression in Lymphatic Tissue, Inflammation, and Cancer
Source: Dis Markers. 2019 Jan 10;2019:5160565. doi: 10.1155/2019/5160565 (PMC6348838; doi:10.1155/2019/5160565)
Supplement: Supplementary 3 — Figure S3: representative images at 100x magnification of a human tonsil and a thyroid gland with Hashimoto thyroiditis stained with serially diluted PD-1 antibody. [file 5160565.f3.pptx]

## Slide 1
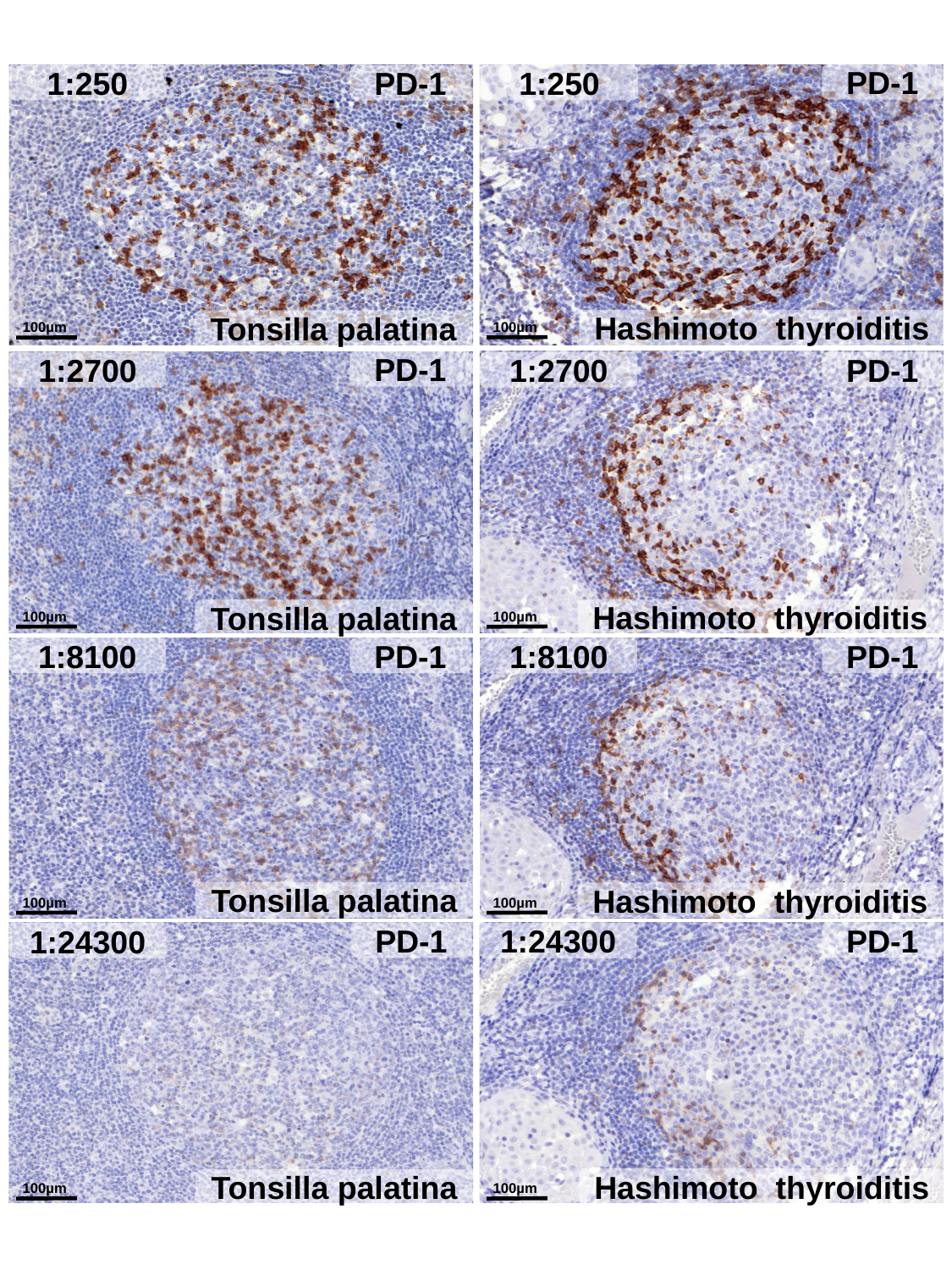

PD-1
1:250
1:250
PD-1
Hashimoto  thyroiditis
Tonsilla palatina
100µm
100µm
PD-1
1:2700
1:2700
PD-1
Hashimoto  thyroiditis
Tonsilla palatina
100µm
100µm
1:8100
1:8100
PD-1
PD-1
Tonsilla palatina
Hashimoto  thyroiditis
100µm
100µm
PD-1
PD-1
1:24300
1:24300
Hashimoto  thyroiditis
Tonsilla palatina
100µm
100µm
Supplementary Figure 1: Serial dilution of the PD-1 antibody in human tonsil and hashimoto thyroiditis to reevaluate the fluorescent results. Supplementary Figure 1: Serial dilution of the PD-1 antibody in human tonsil and hashimoto thyroiditis to reevaluate the fluorescent results.
