# Supplementary figures and images for: Patterns of TIGIT Expression in Lymphatic Tissue, Inflammation, and Cancer
Source: Dis Markers. 2019 Jan 10;2019:5160565. doi: 10.1155/2019/5160565 (PMC6348838; doi:10.1155/2019/5160565)

## Slide 1
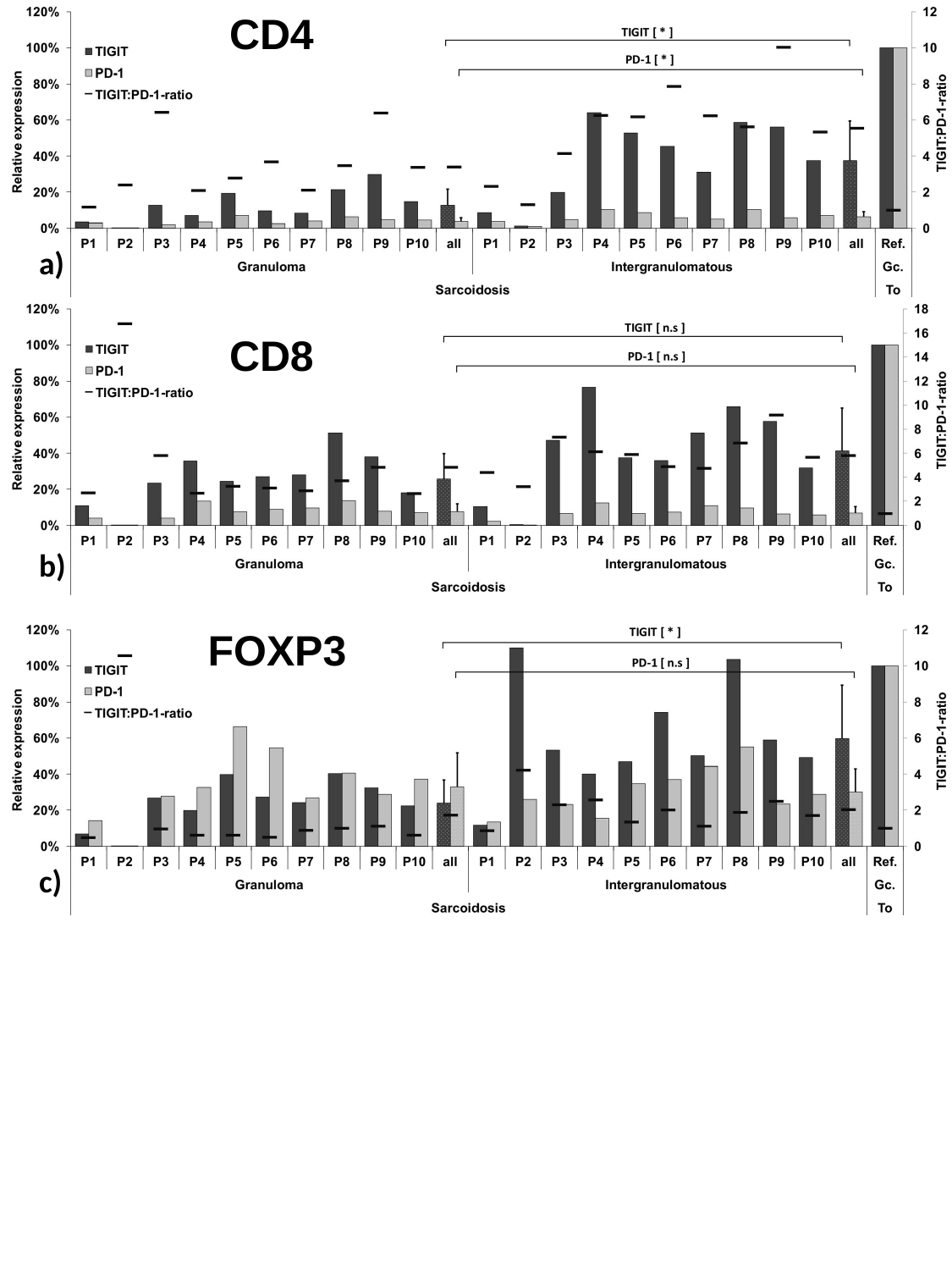

CD4
a)
CD8
b)
FOXP3
c)

Supplement: Supplementary 4 — Figure S4: TIGIT (black) and PD1 (grey) expression levels in (a-c) sarcoidosis and (d-f) Hashimoto thyroiditis. Relative expression refers to the fluorescence measurement in the tonsil germinal centre periphery (Ref. Gc. To.) set to 100%. The black bar shows the TIGIT : PD-1 expression ratio. Gc.: germinal centre; To: tonsil; P: patient. Figure S4 TIGIT (black) and PD1 (grey) expression levels in (a-c) sarcoidosis and (d-f) Hashimoto thyroiditis. Relative expression refers to the fluorescence measurement in the tonsil germinal centre periphery (Ref. Gc. To.) set to 100%. The black bar shows the TIGIT : PD-1 expression ratio. Gc.: germinal centre; To: tonsil; P: patient. [file 5160565.f4.zip › 1/Supplementary Figure 4a_DM_2527093.pptx]

## Slide 1
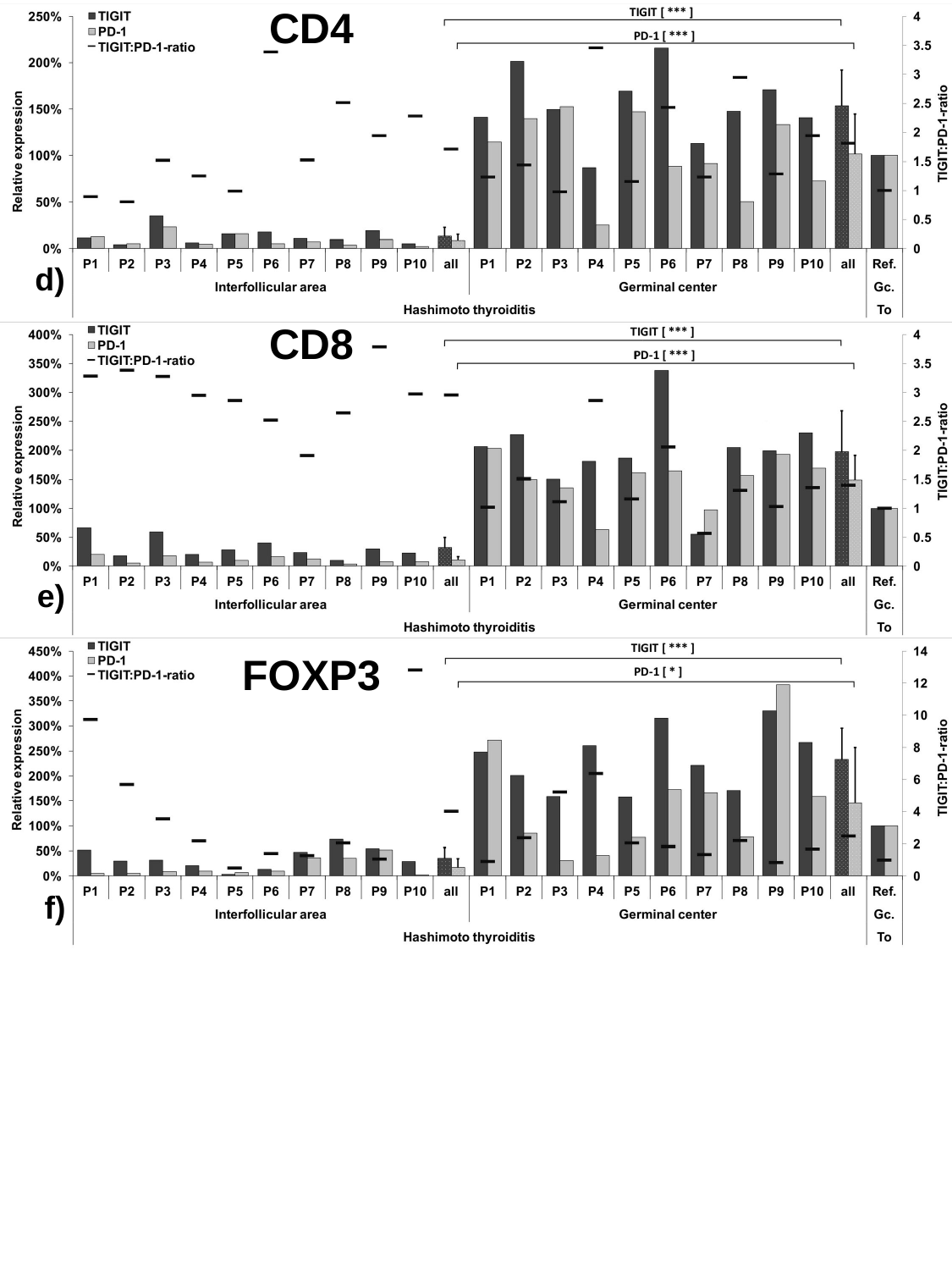

CD4
d)
CD8
e)
FOXP3
f)

Supplement: Supplementary 4 — Figure S4: TIGIT (black) and PD1 (grey) expression levels in (a-c) sarcoidosis and (d-f) Hashimoto thyroiditis. Relative expression refers to the fluorescence measurement in the tonsil germinal centre periphery (Ref. Gc. To.) set to 100%. The black bar shows the TIGIT : PD-1 expression ratio. Gc.: germinal centre; To: tonsil; P: patient. Figure S4 TIGIT (black) and PD1 (grey) expression levels in (a-c) sarcoidosis and (d-f) Hashimoto thyroiditis. Relative expression refers to the fluorescence measurement in the tonsil germinal centre periphery (Ref. Gc. To.) set to 100%. The black bar shows the TIGIT : PD-1 expression ratio. Gc.: germinal centre; To: tonsil; P: patient. [file 5160565.f4.zip › 1/Supplementary Figure 4b_DM_2527094.pptx]
